# Effect of WSP, a Chinese Herbal Formula, on Th17/Treg Ratio and HBeAg Seroconversion in Telbivudine-Treated HBeAg-Positive Chronic Hepatitis B Patients with High Baseline ALT Levels (20–30 Times the ULN)

**DOI:** 10.1155/2019/7260369

**Published:** 2019-03-05

**Authors:** Yang Zhang, Yi-Tong Wang, Jian-Xing Luo, Xiao-Yu Hu, Fang Yang, Wu Lin, Xiao Liang, Bing-Jie Zhao, Song Zhang, Yuan-Yuan Chen

**Affiliations:** ^1^National Integrative Medicine Clinical Base for Infectious Diseases/Department of Infectious Diseases, Hospital of Chengdu University of Traditional Chinese Medicine, Chengdu 610072, Sichuan Province, China; ^2^Clinical Medical College, Chengdu University of Traditional Chinese Medicine, Chengdu 610072, Sichuan Province, China; ^3^School of Basic Medicine, Chengdu University of Traditional Chinese Medicine, Chengdu 610072, Sichuan Province, China; ^4^Department of Neurology, Hospital of Chengdu University of Traditional Chinese Medicine, Chengdu 610072, Sichuan Province, China; ^5^Emergency Department, The Second Affiliated Hospital of Zhejiang University of Traditional Chinese Medicine, Hangzhou 310000, Zhejiang Province, China

## Abstract

Chronic hepatitis B (CHB) is a global health problem. Clinically, many patients have baseline alanine aminotransferase (ALT) levels above 20 times the upper limit of normal (ULN), but there are few reports about these patients. The prospective randomized placebo-controlled clinical study was designed to investigate the effect of WSP, a Chinese herbal formula, on telbivudine- (LDT-) treated HBeAg-positive CHB patients with high baseline ALT levels (20–30 times the ULN) and kidney-yang deficiency syndrome. Eligible patients were randomized to receive LDT 600 mg/day in combination with WSP (treatment group) or placebo granules (control group) 16.28 g/day for 52 weeks. The results showed that HBeAg seroconversion (SC) rate (44.1%) in the treatment group (*n*=34) was significantly superior to that (20.6%) in the control group (*n*=34) at 52 weeks (*P *< 0.05). Meanwhile, WSP could promote HBV DNA negative conversion (85.3%* versus* 61.8%,* P *< 0.05) and ALT normalization (94.1%* versus* 76.5%,* P *< 0.05) compared with the placebo. There were no drug-related serious adverse events. During the treatment, the peripheral blood Th17/Treg ratio first increased and then decreased in the treatment group and reached the peak at 12 weeks (*P* < 0.05). At 12, 24, 36, and 52 weeks, Th17/Treg ratio in the treatment group was better than those in the control group (all* P* < 0.05). In addition, the patients (*n*=22) with HBeAg SC had higher Th17/Treg ratio than the patients (*n*=46) without SC at 12 weeks (0.68±0.26* versus* 0.43±0.18,* P *< 0.001). In conclusion, WSP could safely enhance HBeAg SC and promote HBV DNA negative conversion and ALT normalization in LDT-treated HBeAg-positive CHB patients with high baseline ALT levels (20–30 times the ULN) and kidney-yang deficiency syndrome. Th17/Treg ratio was not only related to the mechanisms of WSP but also a good predictor of 52-week HBeAg SC.

## 1. Introduction 

Hepatitis B virus (HBV) infection is a serious global health problem. It is estimated that 2 billion people worldwide have evidence of past or present HBV infection, and 240 million are chronically infected with HBV [1]. Chronic hepatitis B (CHB) can lead to liver inflammation, which is related to advanced liver diseases including liver failure, cirrhosis, and hepatocellular carcinoma (HCC) [2]. Annually, hepatitis B results in around 650000 deaths, mostly from these complications [1].

Currently, approved anti-HBV drugs include analogs of interferons (IFNs) and nucleos(t)ide analogs (NAs). Relying on their strong viral inhibition, it is easy to achieve the long-term suppression of HBV DNA levels. Hepatitis B e antigen (HBeAg) seroconversion (SC) is a more valuable treatment endpoint for HBeAg-positive CHB in clinical guidelines, associated with long-term prognosis such as lower incidence of cirrhosis and HCC and higher survival rate [1, 3]. Unfortunately, they do not perform well in HBeAg SC. One year of IFNs and NAs treatment only led to about 30–40% and 20% HBeAg SC, respectively [4–9]. Thence, it is necessary to improve HBeAg SC in CHB patients.

Yougui Pill (YGP) is one of the well-known Chinese herbal formulas, documented in Jing Yue Quan Shu (a comprehensive book of traditional Chinese medicine [TCM] from the Ming Dynasty, AD 1640). Based on TCM theory, YGP is a classic formula for warming and tonifying kidney yang, not only for autoimmune encephalomyelitis [10] but also for liver diseases. Modern study [11] has indicated that YGP can inhibit downregulation of MHC II molecule and decrease of adhesion molecule transcription level in dendritic cells induced by glucocorticoid. We further combined these herbs to create a new formula called “Wenshen prescription” (WSP). For many years, WSP was used to treat CHB at the Hospital of Chengdu University of Traditional Chinese Medicine. It turned out that WSP could safely enhance HBeAg SC in telbivudine- (LDT-) treated patients with mild-to-moderate elevation of baseline alanine aminotransferase (ALT) levels (2–10 times the upper limit of normal [ULN]) [12, 13].

In clinical practice, the baseline ALT levels of many patients exceed 20 times the ULN, but there are few reports on the issue of whether to treat these patients immediately or wait until the ALT level drops. The aim of the study was to evaluate the effect of WSP on HBeAg-positive CHB patients with high baseline ALT levels (20–30 times the ULN) and kidney-yang deficiency syndrome.

## 2. Patients and Methods

### 2.1. Patients

In this prospective study, eligible consecutive hospitalized patients with CHB were recruited from the Department of Infectious Diseases, Hospital of Chengdu University of TCM, from July 2013 to May 2014. The clinicians examined all recruited patients and enrolled them into the study according to the criteria of CHB [14]. The inclusion criteria were as follows: (1) 18–60 years of age; (2) hepatitis B surface antigen (HBsAg) positive for at least 6 months; (3) HBeAg positive and anti-HBe negative; (4) 10^5^ copies/mL ≤ HBV DNA level ≤ 10^9^ copies/mL; (5) ALT level between 20 and 30 times the ULN; (6) kidney-yang deficiency syndrome. The kidney-yang deficiency syndrome was confirmed by clinical symptoms and signs manifested as fear of cold, cold of limbs, soreness of waist and knees, pale and enlarged tongue, white coating, deep and thready pulse, or deep and slow pulse. The syndrome must be differentiated by at least two trained doctors of TCM.

Patients were excluded from the study if they had other liver diseases, such as hepatitis A, C, D, E, alcoholic liver disease, autoimmune hepatitis, drug induced hepatitis, Wilson's disease, decompensated liver disease, or other comorbid conditions such as coinfection with HIV, neoplastic disease, severe cardiac or pulmonary disease, psychiatric diseases, therapy with any anti-HBV drug, immunostimulatory and immunosuppressive agents, jaundice (total bilirubin > 3 times the ULN), pregnancy, or breastfeeding. The study protocol was in line with the Helsinki Declaration of 1975 and was approved by the ethics committee of the Hospital of Chengdu University of TCM. Written informed consent was obtained from each patient prior to enrollment.

### 2.2. Preparation of Medicine

WSP was comprised of* Aconitum carmichaelii, Morinda officinalis, Cwscwia australis, Epimedium brevicornum, Astragalus membranaceus, Rehmannia glutinosa, Taxillus chinensis, Scutellaria baicalensis, Artemisia annua, *and* Glycyrrhiza uralensis*, which were listed in Table 1. The placebo consisted of water-soluble starch, glucosum anhydricum, edible chocolate brown pigment, and lyochromes. Both of them were made into granules in Sichuan Neo-Green Pharmaceutical Technology Development Co., Ltd., as the only enterprise in China that had the entire industrial chain and quality control system of TCM Good Agricultural Practice of Medicinal Plants and Animals (GAP) planting base, Chinese herbal piece Good Manufacturing Practice (GMP), and Chinese herbal formula granule development and GMP production.

Every step, from raw materials identification to granules production, strictly followed the standards of Good Manufactory Practice and Chinese Pharmacopoeia [15]. All formula granules are completed in a GMP-certified, fully automated workshop with full computer control. Each Chinese herbal piece was decocted in batches and extracted by decoction extraction methods, which was boiled at 100°C for 60 minutes and 30 minutes, respectively. After extraction, extract was separated, concentrated, and spray-dried into granule form. The chemical composition of the final product was analyzed and all herbs were tested to ensure the safety of human consumption, including heavy metals, microbial contamination, and pesticides. Finally, the various granules were mixed according to their proportion in the Chinese herbal formula and packaged in a sealed plastic box. The composition of a small box of granules (16.28 g) was the same as that of 160 g raw herbs (i.e., daily dose of each patient). The placebo is similar to the TCM granules in shape, color, taste, and packaging.

### 2.3. Study Design

This was a prospective randomized placebo-controlled clinical study. All patients were randomly assigned to a treatment group or control group in a 1:1 ratio. Each patient was instructed to take a small box of granules (16.28 g, either WSP or placebo) for 52 weeks. After the first clinic visit at baseline, patients were required to return at 12, 24, 36, and 52 weeks. Serum HBV DNA, HBeAg and antibody to HBeAg (anti-HBe), HBsAg and antibody to HBsAg (anti-HBs), biochemical tests, CD4 + helper T (Th) 17 and regulatory T (Treg) cells, and adverse events were detected at each clinic visit.

### 2.4. Treatment

Eligible patients were randomized to receive LDT 600 mg/day (Sebivo, Beijing Novartis Pharmaceutical Co., Ltd.) in combination with WSP (treatment group) or placebo granules (control group) 16.28 g/day for 52 weeks. The granules were dissolved in a cup with 200 mL of warm water and taken 100 ml in the morning and afternoon, respectively. Additionally, each patient was given intravenously polyene phosphatidylcholine 930 mg/day (Chengdu Tiantaishan Pharmaceutical Co., Ltd.) and reduced glutathione sodium 1800 mg/day (Kunming Jida Pharmaceutical Co., Ltd.) in hospital for the first 4 weeks and followed up for 48 weeks after discharge.

### 2.5. Laboratory Assays

Patient's laboratory data were uniformly assayed at the Laboratory Department, Hospital of Chengdu University of TCM. Serum HBV DNA was determined by a fluorescent quantifying polymerase chain reaction (PCR) method with a low limit of detection of 1000 copies/mL (Lightcycler-480, Roche, Switzerland). HBV antigens and antibodies were measured by commercially available enzyme immunoassays (Alisei Quality System, RADIM, Italy). Serum ALT, total bilirubin (TBIL), albumin (ALB), creatinine (CREA), and creatine kinase (CK) were detected by a colorimetric method (Automatic Analyzer 7170A, Hitachi, Japan).

The frequency of Th17 cells was detected by flow cytometry. Heparin anticoagulant tube was used to take 5 mL fasting venous whole blood. Peripheral blood mononuclear cells (PBMCs) were separated by Ficoll density gradient centrifugation, and their concentration was adjusted to 2 ×10 ^6^ /mL in RPMI1640 culture solution. The stimulated cells were harvested, centrifuged, resuspended in PBS, and then incubated with 10 *μ*L anti-human CD4-FITC at room temperature away from light for 30 minutes. Following centrifugation and resuspension in 1 mL PBS, cells were added with 100 *μ*L FIX & PERM medium B and incubated away from light for 20 minutes. Then, the treatment group was added with 50 *μ*L anti-human IL-17-PE, while the control group was replaced with 50 *μ*L isotype control monoclonal antibody. Both suspensions were protected from light at room temperature for 20 minutes. Cells were then centrifuged for 5 min, resuspended again in 1 mL PBS, and analyzed by flow cytometry. In addition to adding 10 *μ*L anti-human CD4-FITC, 10 *μ*L anti-human CD25 FITC, and 10 *μ*L FoxP3-PE, the detection method for Treg cells was similar to that of Th17 cells [16].

### 2.6. Endpoints

The primary endpoint was HBeAg SC rate at 52 weeks, and secondary endpoints were the rate of HBV DNA negative (lower than 1000 copies/mL), HBeAg seroclearance, HBsAg seroclearance and SC, ALT normalization (lower than 40 U/L), and Th17/Treg ratio at 52 weeks. In addition, adverse events including symptoms, signs, and clinical laboratory abnormalities (ig, creatine kinase [CK]) within 52 weeks were recorded. According to [17], the CK increase < 7ULN was judged as grade 1/2, and the CK increase > 7ULN was judged as grade 3/4. The date and cause of each patient to withdraw from the study were documented.

### 2.7. Sample Size Estimation

The sample size was calculated based on the data from previous studies [12, 13], which suggested that HBeAg SC rate was 50% in WSP-treated group and 20% in placebo-treated group. The match ratio was 1:1. Assuming a two-sided type I error rate of 5% and a power of 80%, a sample size of 31 in each group was required [18]. On the assumption of a rate of 10% loss to follow-up, 68 target samples were required.

### 2.8. Statistical Analysis

Efficacy and safety analyses were performed according to intention-to-treat (ITT) and performed on the full analysis set (FAS). This set principally included the data from all patients who received at least one dose of the study drugs. Partially missing data of the clinical evaluation were carried forward with the principle of the last visit carried forward (LOCF) [19].

Quantitative data were described by the mean ± SD. Independent-samples* t*-test or Mann-Whitney* U* test was performed to compare differences between quantitative data. Pearson's *χ*2 test, *χ*2 test with continuity correction, or Fisher's exact test was performed to calculate differences between qualitative data. Univariate analysis was used to assess the associations between Th17/Treg ratio and HBeAg SC. All statistical tests were 2-tailed, and a significance level (*P*) of 0.05 was used. The statistical tests were performed using the Statistical Package for the Social Sciences (SPSS version 17.0; SPSS Inc., Chicago, IL, United States).

## 3. Results

### 3.1. Patients

**Figure 1:**
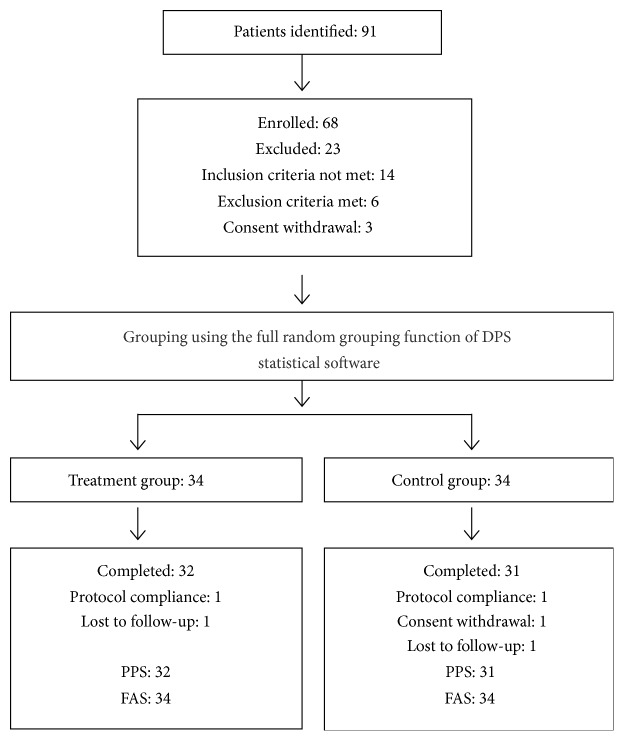
Patients' disposition during the study. PPS, per protocol set; FAS, full analysis set.

Figure 1 shows the patients' disposal during the study. Sixty-eight eligible patients were recruited from 91 CHB patients with high baseline ALT levels. Of them, 63 completed the study, 32 in the treatment group and 31 in the control group. Since all five patients who did not complete the study received treatment and had complete observations on at least one related record at a time point, their clinical data were analyzed by ITT. The FAS population included 68, with 34 in the treatment group and 34 in the control group.

### 3.2. Baseline Characteristics

Table 2 shows the baseline characteristics of patients. There was no significant difference in age, gender, serum ALT, TBIL, PTA, CREA level, Th17/Treg ratio, HBV DNA, and genotype between the treatment group and control group prior to treatment (all* P* > 0.05).

### 3.3. Viral Response

Table 3 shows the effect of WSP on viral and host response. With the treatment duration, the rates of HBV DNA negative, HBeAg seroclearance, and SC in both groups were gradually increased. At 24, 36, and 52 weeks, above indicators were higher in the treatment group than in the control group (all* P* < 0.05). At 52 weeks, 4 patients (4/68, 11.8%) in the treatment group achieved HBsAg seroclearance (*χ*^*2*^=2.391,* P* > 0.05), and 6 patients (6/68, 17.7%) in the control group had virological breakthrough (*χ*^*2*^=4.570,* P *< 0.05). No patient developed primary nonresponse.

### 3.4. Host Response

Compared with patients in the control group, patients in the treatment group had significantly higher ALT normalization rate at 24, 36, and 52 weeks (all* P* < 0.05). During the treatment, the peripheral blood Th17/Treg ratio first increased and then decreased in the treatment group. The ratio reached the peak at 12 weeks, which was significantly higher than the baseline (*P* < 0.05). However, there was no significant difference before and after treatment in the control group (all* P* > 0.05). At 12, 24, 36, and 52 weeks, Th17/Treg ratio in the treatment group was superior to those in the control group (all* P* < 0.05).

### 3.5. Correlation between Th17/Treg Ratio and HBeAg SC

Univariate analysis was used to analyze the relationship between the Th17/Treg ratio and HBeAg SC. After 52 weeks, HBeAg SC was found in 22 patients: 15 patients (15/34, 44.1%) in the treatment group and 7 patients (7/34, 20.6%) in the control group. By comparing the data at each clinic visit, it was found that the patients (*n*=22) with HBeAg SC had higher Th17/Treg ratio than the patients (*n*=46) without SC at 12 weeks (0.68±0.26* versus* 0.43±0.18, t =4.619, P < 0.001).

### 3.6. Safety

No patient developed serious adverse events. All adverse reactions are shown in Table 4, and common adverse reactions were serum CK increases, fatigue, and diarrhea. Thirteen patients in total had serum CK increases: 2 (2/34, 5.9%) in the treatment group and 11 (11/34, 32.4%) in the control group (*χ*^*2*^=7.703,* P* <0.05). Among them, 10 patients had grade 1/2 increases in CK levels: 2 (2/34, 5.9%) in the treatment group and 8 (8/34, 23.5%) in the control group (*χ*^*2*^=4.221,* P* <0.05). There were no obvious abnormalities in ECG, myocardial enzymes (except CK), and echocardiography in patients with CK increases. All adverse reactions are spontaneously relieved.

## 4. Discussion 

In this study, compared with placebo, WSP could safely enhance HBeAg SC and promote HBV DNA negative conversion and ALT normalization in LDT-treated HBeAg-positive CHB patients with high baseline ALT levels (20–30 the times ULN) and kidney-yang deficiency syndrome. Meanwhile, it reduced the incidence of virological breakthrough and CK increases.

To our knowledge, HBV does not kill hepatocytes directly but causes cellular immune response, particularly the activation of HBV-specific cytotoxic T-lymphocytes (CTLs), resulting in virus clearance and hepatocyte damage [20]. If the host develops immune tolerance to HBV, the efficacy of anti-HBV drugs is limited. ALT mainly exists in the cytoplasm, and its activity in serum increases when hepatocytes are damaged. In clinic, ALT is generally considered to indirectly reflect the intensity of cellular immunity in CHB patients. A stratified analysis of baseline ALT levels showed that high baseline ALT levels were independently correlated with increased HBeAg SC rate after either IFNs or NAs therapy [21]. Taking LDT as an example, 32% of patients with baseline ALT levels between 2 and 5 times the ULN and 46% of those with ALT above 5 times the ULN achieved HBeAg SC after 2 years of treatment [22]. Similarly, 37–39% of patients with ALT elevation (2–10 times ULN) [12, 13] and 44% of those with high baseline ALT levels (20–30 times the ULN) achieved HBeAg SC after 1 year of WSP combined with LDT treatment.

However, antiviral treatment for CHB with high baseline ALT levels also may lead to exacerbation. In a Hong Kong study, entecavir was found to be associated with an increase in 48-week mortality in CHB patients with ALT and bilirubin increased beyond 10 and 3 times the ULN, respectively [23]. The authors speculated that entecavir exaggerated immune response, thereby exacerbating liver damage. It is worth discussing whether high baseline ALT levels are an excellent time for antiviral treatment or contraindication. Our study focused on the antiviral effect of WSP on LDT-treated patients with high baseline ALT levels (20–30 times the ULN) and bilirubin below 3 times the ULN, showing that the HBeAg SC rate was 44.1% at 52 weeks, which was significantly higher than LDT alone [4]. More encouragingly, 11.8% (4/34) patients achieved HBsAg seroclearance at 52 weeks in our study. Moreover, it could promote HBV DNA negative conversion and ALT normalization, and no patient developed liver failure. The results are important for treatment strategies of CHB with high baseline ALT levels, especially when IFNs are not available.

CTLs activity is regulated by CD4 T cells [20]. Th17 cells and Treg cells are two newly discovered subtypes of CD4 T cells, derived from the same premise cells. They antagonize each other in cells differentiation and function, and their balance is the key to maintain a proper cellular immune response. Some scholars believed that Th17/Treg ratio may reflect the state of immune imbalance in CHB patients [21]. When CHB patients are in the immune tolerance period, the cytokine TGF-*β* inhibits the inflammatory response by promoting the production of Treg cells. In the immune clearance period, TGF-*β* synergizes with IL-6 or IL-21 to promote the differentiation of Th17 cells and liver inflammation [22]. If the level of IL-6 is decreased, TGF-*β* can promote the expansion of Treg cells and their immune response, while controlling the proinflammatory effect of Th17 cells.

Interestingly, LDT was found to have a higher HBeAg SC rate than other NAs, comparable to IFN [24–27]. It may be that LDT restores the HBV-specific CTLs response. In the present study, Th17/Treg ratio increased first and then decreased gradually. Compared with the control group, Th17/Treg ratio was higher than that of the control group at each observation point other than the baseline. It was demonstrated that WSP could further activate cellular immune response in CHB patients and promote patients to enter the state of immune control with the remission of the disease.

Furthermore, some studies have shown that Th17/Treg ratio is correlated with HBeAg SC [22, 28, 29]. We used a univariate analysis to explore the relationship between Th17/Treg ratio and HBeAg SC. After 52 weeks, a total of 22 patients achieved HBeAg SC: 15 patients in the treatment group (15/34, 44.1%) and 7 patients in the control group (7/34, 20.6%). By comparing the data at each observation point, it was found that the patients (*n*=22) with HBeAg SC had higher Th17/Treg ratio than the patients (*n*=46) without SC at 12 weeks (0.68±0.26* versus* 0.43±0.18,* t*=4.619,* P *< 0.001). It could be seen that Th17/Treg ratio at 12 weeks may be used as a predictor of 52-week HBeAg SC.

In conclusion, WSP could safely enhance HBeAg SC and promote HBV DNA negative conversion and ALT normalization in LDT-treated HBeAg-positive CHB patients with high baseline ALT levels (20–30 the times ULN) and kidney-yang deficiency syndrome. Th17/Treg ratio was not only related to the mechanisms of WSP but also a good predictor of 52-week HBeAg SC. Larger samples and longer-term multicenter studies will lead to a more objective and accurate conclusion. Also, it is critical to investigate whether WSP has similar effect on other NAs and whether multivariate analysis shows other predictors.

## 5. Conclusion

WSP could safely enhance HBeAg SC and promote HBV DNA negative conversion and ALT normalization in LDT-treated HBeAg-positive CHB patients with high baseline ALT levels (20–30 the times ULN) and kidney-yang deficiency syndrome. Th17/Treg ratio was not only related to the mechanisms of WSP but also a good predictor of 52-week HBeAg SC.

## Figures and Tables

**Table 1:** The list of raw herbs composing Wenshen prescription.

Chinese name	Full scientific name	Medicinal parts	Origin	Dose of Chinese herbal piece (g)	Dose after extraction (g)	95% ethanol extract of formula granule (%)	Content of formula granule (mg)
Fu Zi	*Aconitum carmichaelii*	Rhizoma	Liaoning	6	0.48	5.2	
Ba Ji Tian	*Morinda officinalis*	Radix	Guangdong	30	4.25	15.2	
Tu Si Zi	*Cwscwia australis*	Semen	Neimenggu	15	0.88		2.02 (Hyperin)
Yin Yang Huo	*Epimedium brevicornum*	Aerial part	Chongqing	15	0.71		0.70 (Icariin)
Huang Qi	*Astragalus membranaceus*	Radix	Gansu	20	2.86		1.32 (Isoflavone glucoside)
Di Huang	*Rehmannia glutinosa*	Root tuber	Henan	15	2.13		0.28 (Verbascoside)
Sang Ji Sheng	*Taxillus chinensis*	Stem and branch	Yunnan	15	0.71	15	
Huang Qin	*Scutellaria baicalensis*	Radix	Shanxi	30	2.73		3.29 (Baicalin)
Qing Hao	*Artemisia annua*	Aerial part	Chongqing	9	0.82	28.2	
Gan Cao	*Glycyrrhiza uralensis*	Radix and Rhizoma	Gansu	5	0.71		4.26 (Glycyrrhizin) 16.26 (glycyrrhizic acid)
Total				160	16.28		

**Table 2:** Baseline data comparison between treatment and control groups.

Group	Treatment (*n *= 34)	Control (*n *= 34)	*t/χ* ^*2*^	*P *value
Age (yr)	41.26±7.81	43.17±8.20	0.983	0.329
Males (n, %)	25(73.5)	22 (64.7)	0.620	0.431
HBV DNA (log10 copies/mL)	7.18±1.22	7.16±1.27	0.066	0.947
Genotype (B /C/B+C)	17/16/1	19/13/2	0.757	0.686
ALT (U/L)	993.42±151.61	985.67±148.43	0.378	0.707
TBIL (*μ*mol/L)	29.51±12.64	27.40±13.86	0.656	0.514
PTA (%)	78.51±9.98	81.84±16.07	1.026	0.308
Creatinine (*μ*mol/L)	45.73± 22.37	41.03±26.94	0.783	0.437
Th17/Treg cells ratio	0.46±0.14	0.43±0.12	0.949	0.346

All values are expressed as mean ± SD or number (%). HBV, hepatitis B virus; ALT, alanine aminotransferase; TBIL, total bilirubin; PTA, prothrombin activity; Th, T helper cells; Treg, regulator T cells.

**Table 3:** Viral and host responses (*n*, %).

Characteristics	Treatment (*n *= 34)	Control (*n *= 34)	*t/χ* ^*2*^ */z*	*P *value
Viral response				
HBV DNA negative rate				
Week 12	16(47.1)	10(29.4)	2.242	0.134
Week 24	25(73.5)	16(47.1)	4.976	0.026
Week 36	28(82.4)	19(55.9)	5.581	0.018
Week 52	29(85.3)	21(61.8)	4.836	0.028
HBeAg seroclearance rate				
Week 12	3(8.8)	1(2.9)	0.266	0.606
Week 24	11(32.4)	4(11.8)	4.191	0.041
Week 36	15(44.1)	9(26.5)	4.331	0.037
Week 52	21(61.8)	12(35.3)	4.769	0.029
Severity				
HBeAg seroconversion rate				
Week 12	0(0)	0(0)	-	-
Week 24	7(20.6)	1(2.9)	5.100	0.024
Week 36	11(32.4)	4(11.8)	4.191	0.041
Week 52	15(44.1)	7(20.6)	4.300	0.038
Host response				
ALT normalization rate				
Week 12	13(38.2)	8(23.5)	1.722	0.189
Week 24	31(91.2)	21(61.8)	8.173	0.004
Week 36	32(94.1)	23(67.7)	7.703	0.006
Week 52	32(94.1)	26(76.5)	4.221	0.040
Th17/Treg cells ratio				
Week 12	0.63±0.30	0.49±0.20	2.264	0.027
Week 24	0.57±0.26	0.45±0.19	2.173	0.033
Week 36	0.53±0.19	0.42±0.22	2.206	0.031
Week 52	0.44±0.15	0.36±0.14	2.273	0.026

All values are expressed as mean ± SD or number (%). HBV, hepatitis B virus; HBeAg, hepatitis B e antigen; ALT, alanine aminotransferase; Th, T helper cells; Treg, regulator T cells.

**Table 4:** Adverse events of all patients during treatment.

Variable	Treatment (*n *= 34)	Control (*n *= 34)	*χ* ^*2*^	*P *value
Dizziness	0	2	0.515	0.473
Headache	1	0	0.000	1.000
Diarrhea	2	1	0.000	1.000
Fatigue	1	3	0.266	0.606
Nausea	0	1	0.000	1.000
Rash	0	1	0.000	1.000
Serum CK increase	2	11	7.703	0.006
Grade 1/2	2	8	4.221	0.040
Grade 3/4	0	3	1.395	0.238

## Data Availability

The data used to support the findings of this study are included within the article.
